# Golden hour of neonatal life: Need of the hour

**DOI:** 10.1186/s40748-017-0057-x

**Published:** 2017-09-19

**Authors:** Deepak Sharma

**Affiliations:** National Institute of Medical Science, Jaipur, Rajasthan India

**Keywords:** Golden hour, Neonate, Preterm, Term

## Abstract

“Golden Hour” of neonatal life is defined as the first hour of post-natal life in both preterm and term neonates. This concept in neonatology has been adopted from adult trauma where the initial first hour of trauma management is considered as golden hour. The “Golden hour” concept includes practicing all the evidence based intervention for term and preterm neonates, in the initial sixty minutes of postnatal life for better long-term outcome. Although the current evidence supports the concept of golden hour in preterm and still there is no evidence seeking the benefit of golden hour approach in term neonates, but neonatologist around the globe feel the importance of golden hour concept equally in both preterm and term neonates. Initial first hour of neonatal life includes neonatal resuscitation, post-resuscitation care, transportation of sick newborn to neonatal intensive care unit, respiratory and cardiovascular support and initial course in nursery. The studies that evaluated the concept of golden hour in preterm neonates showed marked reduction in hypothermia, hypoglycemia, intraventricular hemorrhage (IVH), bronchopulmonary dysplasia (BPD), and retinopathy of prematurity (ROP). In this review article, we will discuss various components of neonatal care that are included in “Golden hour” of preterm and term neonatal care.

## Introduction

**Fig. 1:**
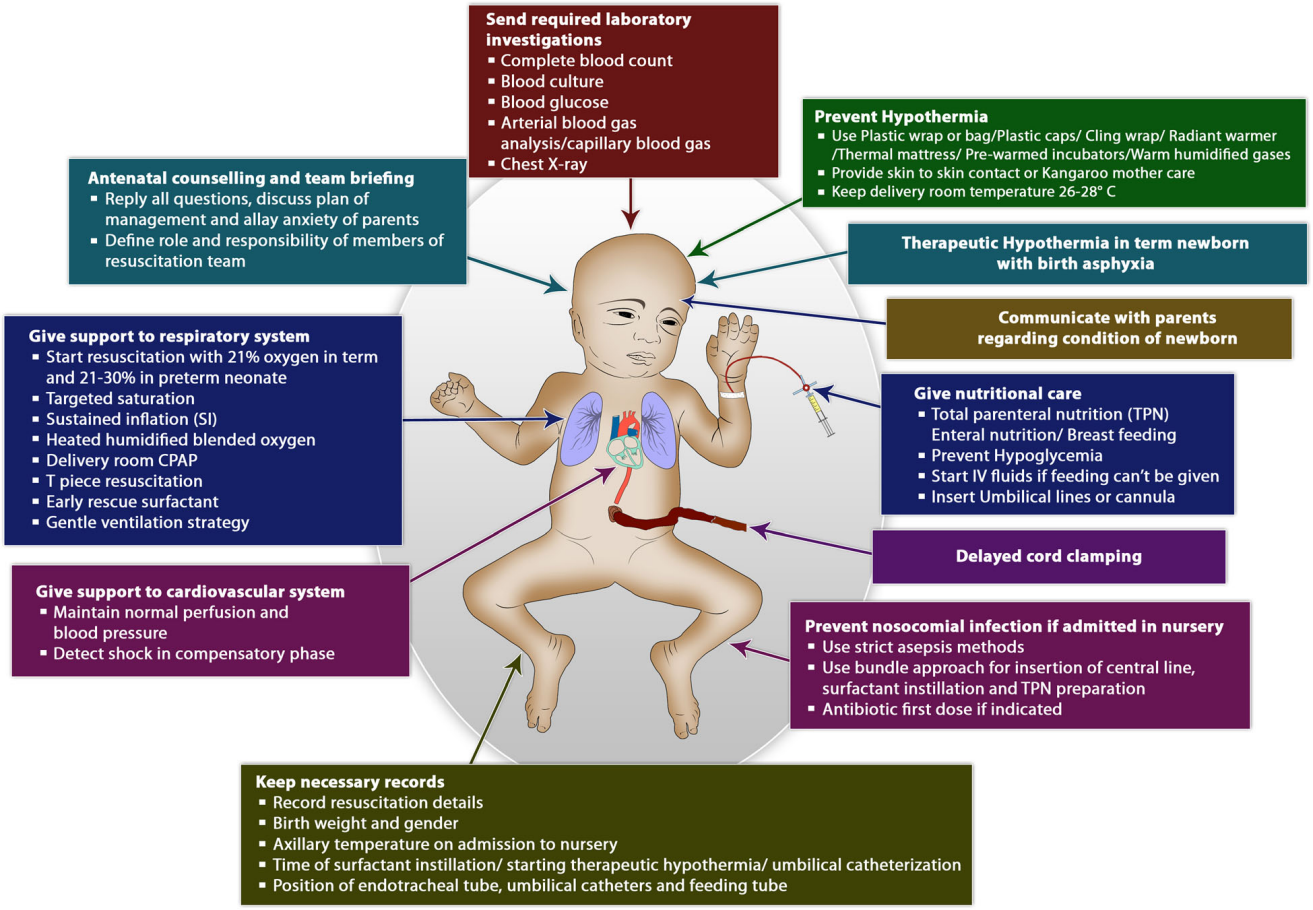
Figure showing golden hour interventions to be done at the time of preterm and term newborn birth (Figure copyright Dr Deepak Sharma)

The concept of “Golden Hour” has been introduced recently in field of neonatology, highlighting the importance of neonatal care in the first 60 minutes of postnatal life [1]. The golden hour term has been adopted from adult trauma where it is used for the initial first hour of trauma management [2, 3]. Dr. R. Adams Cowley gave the concept of “Golden Hour” in emergency medicine and showed that with the use of golden hour approach there was decrease in patient mortality with better transport and patient outcome [2, 4]. Reynolds et al. was the first person to implement this concept in the neonatal care [1]. The neonatal management in the first hour of life have an important effect on both immediate and long-term outcomes of all neonates. There are many interventions that needs to be practiced in golden hour for neonatal care so that neonatal complications are minimized [5]. The prime objective of golden hour is to use evidence based interventions and treatment for better neonatal outcome, importantly for extremely low gestational age neonates (ELGAN) [6]. In the golden hour, standard approach is followed derived from the best available evidence with aim of practicing gentle but timely and effective interventions with non-invasive procedures if required [7]. In this review article, we have covered the various components of golden hour approach in preterm and term neonatal care (Fig. 1 and Table 1). The details of all the interventions with current evidence can be read from other published reviews of the author [8, 9].

**Table 1:** Various components of “Golden 60 minutes” project for term and preterm newborn

S. no	Components
1	Antenatal counseling and team briefing
2	Delayed cord clamping
3	Prevention of hypothermia/temperature maintenance
4	Support to respiratory system
5	Support to cardiovascular system
6	Early nutritional care
7	Prevention of hypoglycemia
8	Initiation of breast feeding
9	Infection prevention
10	Starting of therapeutic hypothermia for birth asphyxia
11	Laboratory investigation
12	Monitoring/record
13	Communication with family

### Antenatal counseling and team briefing

Infants born at an extremely low gestational age have a high mortality rate and are at risk of having neurodevelopmental disabilities ranging from subtle to severe in grade [10–12]. Estimated gestational age of delivery has shown strong association with neurodevelopmental outcome and it serves as the basis for antenatal counselling [11], although it has some fallacies that limits its role for using as single parameter like the rate of fetal development during the early third trimester and the inaccuracy of gestational age dating. The goal of antenatal counseling is to inform parents and assist them in decision-making over either providing resuscitation or giving only comfort care to the neonate. The parents can be offered comfort care for the newborn born at gestational below 25 weeks, but this decision needs to be made after consideration of region specific guidelines. The decision to provide comfort care will also depend upon the variables such as perceived accuracy of gestational age assignment, the presence or absence of chorioamnionitis, and the level of care available for location of delivery [13–15]. The three components of effective counseling are assessment of risks, communication of those risks, and ongoing support. Antenatal counselling especially before a preterm delivery has many benefits for parents like reducing parental anxiety, increasing knowledge, facilitating informed decision-making and making rapport with the neonatal clinicians [16, 17]. In the counselling the parents should be told about most accurate prognostic morbidity and mortality data available for their infant based on hospital-specific database or regional or national data [18, 19]. Parents also need to be informed that inspite to the best efforts, both things namely the exact prognosis or outcome for a specific infant either antenatally or immediately after delivery and prediction of long-term neurologic outcome remains limited [20, 21]. The parents should be given appropriate time for making decision over the neonatal management [17, 22]. Evidence shows that rational, consensus periviability guidelines are well accepted and can be used for antenatal counselling by neonatologists, obstetricians, and nurses who provide care to pregnant women and infants at extremely low gestational ages [23].

The importance of antenatal counselling has been emphasized in neonatal resuscitation programme (NRP) 2015. The parents should be counselled antenatally, answering all the question asked by them about the newborn thus allaying there anxiety. This hold true for both pre-term and term neonate. If the newborn is going to be shifted to mother side than parents need to be counselled regarding breast feeding and general newborn care [13, 14] The parents should also be told about expected duration of neonatal intensive care unit (NICU) stay and morbidities which the newborn is likely to face in the postnatal life depending upon the gestational age and birth weight. The prediction of hospital stay duration will depend upon the gestational age at birth, expected morbidities and previous database of the hospital for the same gestational age neonate. The pregnancy outcome will depend upon the birth weight, gestational age, gender, use of prenatal steroids, singleton pregnancy, maternal age, maternal health, maternal nutrition, substance use, genetics, and complications during pregnancy [17, 19, 22]. The challenges of antenatal counselling includes counselling for near viability birth and fetus having antenatally diagnosed malformations. If the fetus is diagnosed with congenital malformations (e.g. congenital diaphragmatic hernia, abdominal wall defects, lung malformations, tetralogy of Fallot, hydronephrosis, or sacrococcygeal teratoma) pediatric surgeon should be brought in picture and the goal of counselling should be to inform parents regarding the implications of the fetal anomaly, reduce parental anxiety and stress and inform about the post-natal intervention like need for ventilation, hemodynamic stabilization and surgical intervention in such cases. When the fetus is diagnosed with immune hydrops fetalis than parents need to be counselled for exchange transfusion after birth and need for storage of blood antenatally so that management of the neonate is not delayed. The outcome of these neonates in such cases will depend upon the other associated malformations, lung: head ratio in case of diaphragmatic hernia, and severity of pulmonary stenosis in case of tetralogy of Fallot [24]. The parents should be counselled about the possible outcome (mortality and morbidity) keeping the past record of such newborn in the hospital [25]. Ideally both obstetrics team and neonatal team should do counselling together and present a consistent approach for the newborn as this leads to better rapport between neonatologist, obstetrician and parents.

When called for attending birth of high risk neonate than the resuscitation team should decide for the team leader and every member of the team should be given role before the delivery of the neonate so that during resuscitation there are no confusions over interventions and and thus avoiding any miss-happening. The NICU personal need to be informed for the expected neonatal admission, especially if the team is going to attend birth of any preterm neonate or high-risk term neonate. Working condition of the instruments needed during resuscitation should be checked and should be in adequate number if twins or triplets are being expected. Pre-resuscitation check list use make this equipment checking process very easy and rapid. Maternal history should be read in detail from the maternal records and required details should be noted down. The team who is going to attain the delivery of extreme premature, or term neonate with malformations (hydrops fetalis, congenital diaphragmatic hernia, upper airway malformation), should be expertise in attending such neonates and should have necessary skills for all interventions that may be required in the delivery room. During the time neonate reaches NICU, neonatal bed should be made ready and all required medications should be procured, thus avoiding delay in treatment once the shifting process is over [26, 27].

### Delayed cord clamping (DCC)

Physiologically early cord clamping (ECC) have been defined as clamping of cord when still there is circulation of blood from placenta to newborn and delayed cord clamping (DCC) has been defined as clamping of cord after stoppage of placental circulation [28, 29]. When timing of cord clamping is taken in consideration for the definition of ECC and DCC, then before the mid-1950s cord clamping within one minute after birth was termed as ECC, and clamping after five minutes was defined as DCC [30]. The present studies that compared ECC and DCC, have defined ECC as cord clamping immediately or within 15 seconds of birth and DCC as cord clamping after 30 seconds to 3 min after neonatal birth [31–33]. The fetal-placental circulation contains blood that approximates to 110 -115 mL/kg of fetal body weight, with approximately 35 - 40% of total is present in the placenta at one point of time [34, 35]. In term newborn, DCC for one-minute lead to transfer of 80 ml extra blood and delay of three minutes leads to total transfer of 100 ml blood to the neonate [36]. The factor that determine the placental transfusion includes cord clamping time, uterine contractions, umbilical blood flow, newborn respiration and gravity [37]. The placental flow to fetus decrease rapidly after the neonatal birth and after three minutes of birth, placental blood flow to neonate becomes insignificant, and by 5 minutes it absolutely ceases [38]. NRP 2015 guideline recognizes the importance of DCC in newborn and thus included new recommendations over this issue. It recommends DCC clamping (delay in cord clamping for more than 30 seconds) for all preterm and term newborn not requiring resuscitation at birth. The guidelines also further states that in newborn that requires resuscitation at time of birth, DCC should not be done till further trials are done seeking the feasibility over this [13, 14]. The author inference from the available evidence is to delay the cord clamping for more than 30 seconds after the birth and should not be practiced in newborn requiring resuscitation. Newer beds equipped with neonatal resuscitation equipments are being made to implement the concept of DCC in newborn requiring resuscitation, although trials are still needed to see feasibility of such beds during neonatal resuscitation [39]. Cochrane meta-analysis studied the effect of timing of umbilical cord clamping and other strategies to influence placental transfusion in preterm birth on maternal and infant outcomes. This meta-analysis included fifteen studies and showed that DCC was associated with fewer infants requiring transfusions for anaemia (RR 0.61, 95% CI 0.46 - 0.81), less intraventricular haemorrhages (IVH) (ultrasound diagnosis all grades) (RR 0.59, 95% CI 0.41- 0.85) and lower risk for necrotizing enterocolitis (NEC) (RR 0.62, 95% CI 0.43 - 0.90) compared with ECC. The peak bilirubin concentration was significantly higher in neonates allocated to DCC compared with ECC (mean difference 15.01 mmol/L, 95% CI 5.62 - 24.40) [40]. The Cochrane meta-analysis that sought the effect of DCC in term neonates reported no significant differences in neonatal mortality, Apgar score < 7 at five minutes and neonatal admission. There was significant increase in mean birth weight in DCC (101 grams; 95% CI 45 – 157), although neonates in DCC required more phototherapy for neonatal jaundice (RR 0.62; 95% CI 0.41- 0.96). The haemoglobin concentration in infants at 24 to 48 hours was significantly higher in DCC group [41]. Thus the current evidence shows various beneficial effect of DCC except significant increase in requirement of phototherapy. There is still controversy over the correct time for DCC with many unresolved question and clinical issues for its successful implementation [30, 42].

There are concerns about practicing DCC in extreme premature and neonates requiring resuscitation, for which umbilical cord milking (UCM) has emerged as an alternative. In the umbilical vein alone there is approximately 15 to 20 mL of cord blood which can be transferred to newborn with cord milking [43]. UCM is practiced by milking 20 cm of umbilical cord 2 to 3 times before clamping at a rate of 20 cm per 2 seconds while the infant is held at or below the level of the placenta [44, 45]. The NRP 2015 guidelines are against the routine use of cord milking for newborn who are born at < 29 weeks of gestation except in a research setting [13, 14].

### Prevention of Hypothermia

Hypothermia defined as temperature < 36.5 °C is a dangerous problem in newborn especially in very low birth weight (VLBW), extremely low birth weight (ELBW) and ELGAN. The reported incidence of hypothermia at time of admission to the NICU in VLBW newborn ranges from 31% to 78% [46–49]. There is 28% increase in neonatal mortality with each 1 °C fall in axillary temperature while admitted in NICU [46], and admission temperature in NICU is a strong predictor for neonatal mortality [50, 51]. The highest risk of neonatal hypothermia is within the first minutes to hours after birth as there is wide difference between in-utero and environmental temperature [52]. After the birth of newborn, there is rapid loss of heat by four mechanisms namely conduction, convection, evaporation and radiation. The neonate develop hypothermia because of larger surface area per unit body weight, high surface area to volume ratio, increased evaporative fluid losses from the skin, very large surface area of head compared to body, and presence of thin layer of subcutaneous fat thus making them susceptible to easily develop hypothermia [53, 54]. Hypothermia leads to significant increase in Apgar score less than 7, IVH, late onset sepsis, hypoglycemia and respiratory distress [50]. NRP 2015 guideline recommend maintaining temperature between 36.5 to 37.5 °C and avoiding hyperthermia (>37.5 °C) [13]. Hypothermia can be prevented by keeping delivery room temperature from 26 to 28 °C, using pre-warmed linen sheets to receive the newborn just after birth, re-warming surfaces and eliminating drafts. In the delivery room and during transportation various interventions that can be practiced to prevent hypothermia are using plastic wrap or bag [55, 56], plastic caps, cling wrap, radiant warmer, thermal mattress [57, 58], pre-warmed single/double walled incubators [59], warm humidified gases [60, 61], and skin to skin contact [59, 62, 63]. The preterm newborn just after the birth is covered with polyethylene wrap/cling wrap or transferred into vinyl bag without drying with all necessary resuscitation steps being carried out with newborn covered in wrap. The wrap/bags is to be removed only after the newborn is shifted to nursery and is stabilized [64–70]. Plastic wraps/cling wraps/plastic caps/vinyl bags prevents heat loss by evaporation, radiant warmer prevent heat loss secondary to radiation and conduction, incubator prevent heat loss by conduction, convection, radiation and evaporation, whereas skin to skin contact and warm humidified gases acts by preventing conductive heat loss. The insensible fluid losses can be decreased by placing the infant in a double walled incubators like giraffe/ humidified isolette on arrival to NICU with humidification of 70-80% [71]. NRP 2015 recommend the use of polyethylene wrap for prevention of hypothermia and early skin to skin contact in term newborn [13, 72]. The term neonate who are stable can be dried in delivery room and hat can be placed to prevent evaporative heat loss [73]. In lower and middle income countries, NRP 2015 recommend use of clean food-grade plastic bag up to the level of the neck and skin to skin contact to prevent hypothermia [13, 14]. Cochrane meta-analysis reported plastic wraps or bags, plastic caps, skin-to-skin care (SSC) and trans-warmer mattress being effective in reducing heat losses and reducing hypothermia [73].

### Support to respiration

The goal of providing support to respiratory system is to help in the smooth transition of gas exchanging organ from placenta to lung. Support to respiratory system forms an important part of golden hour management. Both preterm and term newborn are prone to develop respiratory distress immediately after birth. Although the etiology of this respiratory distress could be varied, but the immediate goal of the neonatologist in delivery room should be in providing support to the respiratory system of these newborns [5]. NRP 2015 recommended that resuscitation of preterm newborns (< 35 weeks of gestation) should be started with 21 to 30% oxygen, and in newborn > 35 weeks of gestation, resuscitation should be started with room air. Use of pulse oximetry is recommended when resuscitation is anticipated, when positive pressure ventilation (PPV) is administered, when central cyanosis persists beyond the first 5 to 10 minutes of life, or when supplementary oxygen is administered. The goal of oxygen therapy is to achieve pre-ductal oxygen saturation as per the time specific interquartile range recommended in NRP (1 min 60-65%, 2 min 65-70%, 3 min 70-75%, 4 min 75-80%, 5 min 80-85% and 10 min 85-95%) [13, 14] When neonates are transported to NICU and started on invasive mode than targeted saturation should be 90-95% in the preterm infants [74–77].

As per the latest NRP 2015, newborn who are born with meconium stained amniotic fluid and are vigorous (defined as having a normal respiratory effort and normal muscle tone) should be provided routine care and the infant may stay with the mother. If the newborn is not vigorous (defined as having depressed respiratory effort and poor muscle tone), the initial steps of resuscitation should be completed under the radiant warmer. Routine tracheal intubation for tracheal suctioning in non-vigorous neonate is not recommended and PPV should be initiated if the infant is not breathing or the heart rate is less than 100/min after the completion of initial steps. During neonatal resuscitation, endotracheal intubation is performed when bag-mask ventilation is ineffective or prolonged, when chest compressions are "anticipated" or preferably prior to onset of chest compressions, or when bag and mask ventilation is contraindicated such as congenital diaphragmatic hernia [13, 14]. Exhaled/End tidal CO2 detection is the most reliable method of confirmation of endotracheal tube placement [78, 79]. CO2 detector are of two type namely colorimetric devices that change color in the presence of CO2 (colour changes from blue/purple to yellow) and capnographs that are electronic monitors displaying the CO2 concentration with each breath and these detectors are attached to the ET tube. It gives false result when the infant is in cardiac arrest leading to unnecessary extubation and reintubation in critically ill newborns [80]. If there is no improvement in heart rate after effective resuscitation, chest compression and drugs or there is sudden deterioration of any infant on ventilation than suspicion of pneumothorax is kept. Transillumination test can be done in delivery room for detection of air leak syndrome [81]. If a pneumothorax causes significant respiratory distress, bradycardia, or hypotension, it should be relieved urgently by placing a catheter into the pleural space and evacuating the air. If the baby has ongoing respiratory distress, in the NICU thoracostomy tube can be inserted and may be attached to continuous suction. Pneumothorax can be prevented by avoiding giving too much inflating pressure and high PEEP during resuscitation [13, 14]. If there is suspicion of pulmonary hypoplasia than such infants should be started on invasive ventilation with goal of gentle ventilation and hemodynamic monitoring [24].

The aim of providing early respiratory support is to achieve functional residual capacity (FRC), provide appropriate tidal volume (4 -6 ml/kg) and minute ventilation, decrease work of breathing, avoid apnea, and to avoid invasive ventilation by providing assistive ventilation. The respiratory system can be supported by sustained lung inflation (SLI) [82–86], delivery room continuous positive airway pressure (CPAP) [87, 88], blended heated humidified oxygen, use of T piece resuscitation for providing PEEP (Peak end expiratory pressure) and PIP (Peak inspiratory pressure), early rescue surfactant (avoiding prophylactic surfactant) [75, 89–91], and use of gentle ventilation strategies [volume guarantee [92], patient triggered ventilation, permissive hypercapnia (tolerating PCo2 upto 55- 60 mmHg provided the pH >7.2) [93, 94], targeted saturation (90-95%) in preterm [75–77], avoiding hypoxia and hyperoxia, proper humidification, early extubation and using non-invasive modes of ventilation like CPAP and non-invasive mechanical ventilation (NIMV)] [95–99]. The goal of providing PPV is to use just enough pressure to inflate and aerate the lungs so that the heart rate and oxygen saturation increase. In the delivery room we can start PPV with PIP of 20 to 25 cm H2O [Full-term babies may require a higher PIP for the first few breaths to inflate their lungs (30-40 cm H2O)], PEEP of 5 cm H2O, respiratory rate of 40- 60 breaths/minute and FiO2 of 21% in ≥ 35 weeks and 21-30% in < 35 weeks gestational age newborn. The most important indicator of successful PPV is a rising heart rate. The adequacy of PIP is see as gentle rise and fall of the chest with each breath. If there is no chest rise than ventilation corrective steps should be done (MRSOPA=Mask adjustment, Reposition head, Suction airway, Open mouth, Pressure increase, and Alternative airway). After starting of effective PPV, monitor the baby’s chest movement, heart rate, and respiratory effort. FiO2 need to be adjusted based on pulse oximetry. When the heart rate is consistently more than 100 bpm, gradually reduce the rate and pressure of PPV, observe for effective spontaneous respirations, and stimulate the baby to breathe. PPV may be discontinued when the baby has a heart rate continuously over 100 bpm and sustained spontaneous breathing. After PPV is stopped, there is need for continues monitoring of the newborn oxygen saturation and breathing. Free-flow oxygen or CPAP may be required and can be weaned, as tolerated, based on pulse oximetry. PPV can be provided by self-inflating bag, flow-inflating bag and T piece resuscitator. T piece resuscitator requires a compressed gas source and pressure adjustment to deliver PIP and PEEP, hence need to be used in delivery room that have source of compressed gas. Its primary advantage is that it provides more consistent pressure with each breath than the self-inflating bag. It need to be use when there is need to deliver CPAP to a spontaneously breathing newborn, providing SLI and when there is need to deliver PIP also. Self-inflating bag doesn’t need any source of compressed gas, hence can be used in resuscitation where there is no availability of compressed gas [86].

Sustained inflation (SLI) strategy leads to lung recruitment immediately after birth through delivery of brief peak pressure to the infant airways via a nasopharyngeal tube or mask, thus allowing preterm infants to achieve FRC. SLI leads to better alveolar recruitment, increase in pulmonary blood flow and decrease in pulmonary vascular resistance, movement of lung liquid out of the alveoli, and uniform lung expansion and better compliance [86]. After oropharyngeal and nasal suctioning, pressure-controlled (20-30 cmH_2_O) inflation is sustained for 5-15 seconds, using a neonatal mask and a T-piece ventilator, followed by the delivery of 5 cmH_2_O NCPAP. Patients is observed for the following 6 to 10 seconds to evaluate their cardio-respiratory function. If respiratory failure persists (that is, apnea and gasping) and/or the heart rate is >60 and <100 bpm despite NCPAP, the SLI maneuver (again 20-30 cmH_2_O for 5-15 seconds) is repeated. If the heart rate remains >60 and <100 bpm after the second SLI maneuver, the infant is resuscitated following the current guidelines of the NRP [82, 83, 85, 100, 101]. The European Resuscitation Council Guidelines recommend SI for the initial ventilation of apneic term and preterm infants [102].

In a ventilated infant Oxygen saturation index (OSI = Mean airway pressure × FiO2 × 100 : SpO2) is an non-invasive method to assess severity of hypoxic respiratory failure, acute respiratory distress syndrome or acute lung injury [103]. OSI of 6.5 equals to the acute lung injury criteria, and an OSI of 7.8 equals to acute respiratory distress syndrome criteria [104]. The goal of invasive ventilation should be minimizing lung injury secondary to ventilation namely barotrauma, volutrauma, atelectotrauma, biotrauma and rheotrauma [92, 94, 105]. Volume targeted mode of ventilation has shown to cause reduction in the combined outcome of death or BPD, pneumothorax, hypocarbia and the combined outcome of periventricular leukomalacia or grade 3-4 IVH, thus making it a preferred mode over pressure control mode in neonatal ventilation [92, 106]. When nasal CPAP is compared with nasal intermittent positive pressure ventilation (NIPPV) as primary mode of respiratory support, NIPPV has found to superior to NCPAP for decreasing respiratory failure and the need for intubation and endotracheal tube ventilation among preterm infants with respiratory distress syndrome (RDS) [107]. Similarly for post-extubation respiratory support, NIPPV has shown to be superior over NCPAP in reducing the incidence of extubation failure and the need for re-intubation within 48 hours to one week [108]. Any wrong intervention done in golden hour leads to damage of the lung parenchyma, and plays an important role in development of bronchopulmonary dysplasia (BPD) in ELBW, VLBW or EGLAN [109].

Preterm neonates are born with less surfactant pool and the use of surfactant in the management of respiratory distress syndrome has revolutionized care of the preterm infants. Animal derived surfactant is preferred over protein free synthetic surfactant as it leads to greater early improvement in the requirement for ventilator support, fewer pneumothoraxes, and fewer deaths [110]. When protein containing synthetic surfactants was compared with animal derived surfactant, results of the study showed equal efficacy [111]. Surfactant should be given as “early rescue” i.e. within two hours of neonatal birth and is preferred over “late rescue” with simultaneous respiratory system support in form of either invasive ventilation or non-invasive ventilation depending upon the clinical condition of the infant [112]. The indication for giving surfactant are preterm infants born at <30 weeks’ gestation who need mechanical ventilation because of severe RDS and infant showing signs of RDS and need more than 30% inspired oxygen to maintain saturations in the normal range [75]. Surfactant should be administered in the standard method of aliquots instilled into an endotracheal tube. There are multiple method of surfactant administration namely administration through catheter, side port, or suction valve; administration through dual-lumen endotracheal tube; administration through a laryngeal mask airway; nasopharyngeal administration of surfactant and INSURE. INSURE (INtubation SURfactant administration and Extubation to CPAP) is the preferred method of surfactant installation if the infant has good respiratory efforts [113, 114]. The complications of surfactant administration includes transient airway obstruction, oxygen desaturation, bradycardia, and alterations in cerebral blood flow and brain electrical activity [115]. Early surfactant replacement therapy with extubation to NCPAP compared with later selective surfactant replacement and continued mechanical ventilation with extubation from low ventilator support has shown to be associated with less need mechanical ventilation, lower incidence of BPD and fewer air leak syndromes [116]. With the use of delivery room CPAP, using “prophylactic surfactant” i.e. surfactant installation within 15 minutes of birth is not recommended in clinical practice [91]. Recently there has been research in less invasive surfactant administration (LISA) and studies have shown that it leads to lower rates of mechanical ventilation, postnatal steroids, BPD and BPD or death than the controls [117–119].

Airway obstruction is a life-threatening emergency and needs immediate management. The newborn’s airway may be obstructed by thick secretions or a congenital anomaly that leads to an anatomic obstruction. If newborn with Pierre robin sequence develops labored breathing, he is turned prone on his stomach. In this position, the tongue move forward and open the airway. If prone positioning is not successful, small endotracheal tube (2.5 mm) is inserted through the nose with the tip placed deep in the posterior pharynx, past the base of the tongue, and above the vocal cords. It is not inserted into the trachea and there is no requirement of laryngoscope. This helps in relieving the airway obstruction. In these newborn who develop severe difficulty in breathing and requires resuscitation, face-mask ventilation and endotracheal intubation may be very difficult and laryngeal mask may provide a lifesaving rescue airway. In newborn choanal atresia is usually unilateral and does not cause significant symptoms in the newborn period. Newborn with bilateral choanal atresia may develop respiratory distress immediately after birth. The mouth and airway can be kept open by inserting one of the following into the baby’s mouth—a feeding nipple or pacifier modified by cutting off the end (McGovern nipple) and secured with ties around the occiput, an oral endotracheal tube positioned with the tip just beyond the tongue in the posterior pharynx, or a plastic oral (Guedel) airway. In newborn with congenital high airway obstruction (CHAOS), then there is need for special expertise and equipment for successful intubation. If the obstruction is above the level of the vocal cords than placement of a laryngeal mask may provide a lifesaving rescue airway. If the obstruction is below vocal cords than there will need for emergency tracheostomy, therefore if CHAOS is diagnosed antenatally than such babies should be born in a facility where emergency management of the airway by a trained multidisciplinary team is immediately available in the delivery room [13, 14].

The clinical course of respiratory distress also helps in differentiating the various cause of distress. In RDS there is onset of respiratory distress immediately after birth followed by gradual worsening of distress over next six hours. In air leak syndrome there will be sudden onset of hypoxia and hypercarbia, chest hyperinflation and reduced air entry. In neonate with PPHN there will be severe cyanosis, extreme liability especially on handling, tricuspid systolic murmur and differential saturation of pre-ductal and post-ductal. In MAS there will cord stained of meconium in term or post-term neonate, tachypnea, hypoxia, barrel shaped chest and PPHN. Chest X-ray done in NICU will also further help in diagnosing the etiology of respiratory distress [120, 121].

### Support to cardiovascular system

The goal of giving support to cardiovascular system is to have normal capillary refill time (For 5 seconds, finger is pressed over sternum to have blanching of skin and then count seconds to see disappearance of blanching, consider abnormal when it is ≥ 3 second), heart rate and blood pressure in both preterm and term newborn [122]. The first parameter that shows effectiveness of resuscitation is improvement in heart rate. To assess the response to initial steps of resuscitation, auscultation along the left side of the chest is the most accurate physical examination method of determining a neonate’s heart rate. Estimation of the heart rate is done by counting the number of beats in 6 seconds and multiplying by 10. If the newborn requires PPV during resuscitation than assessment of heart rate is done using 3 lead ECG, which is more reliable than pulse oximetry for detection of heart rate. Thus as per NRP 2015 during resuscitation of term and preterm newborns, the use of 3-lead ECG is recommended for the rapid and accurate measurement of the newborn’s heart rate [13, 14]. The interventions that may be needed in delivery room for supporting cardiovascular system includes bag and mask ventilation, intubation and PPV, chest compression and rarely drugs (normal saline and adrenaline). Venous access should be established at the earliest with umbilical vein being the easiest vein to be cannulated through which medicines are given. Umbilical venous catheter (UVC) is inserted for 2 to 4 cm (less in preterm babies) until there is free flow of blood [13]. The medicine are required during resuscitation when inspite of effective ventilation and chest compression, the newborn still have a heart rate below 60 bpm. The medicine required during resuscitation are epinephrine and normal saline (0.9% NaCl). Epinephrine is given if the newborn heart rate remains below 60 bpm after 1) At least 30 seconds of PPV that inflates the lungs (moves the chest), and 2) Another 60 seconds of chest compressions coordinated with PPV using 100% oxygen. The recommended intravenous or intraosseous dose is 0.1 to 0.3 mL/kg (equal to 0.01 to 0.03 mg/kg) for 1:10,000 dilution. If epinephrine is to be given by endotracheal pathway, than the recommended dose is 0.5 to 1 mL/kg (equal to 0.05 to 0.1 mg/kg) for 1: 10,000 dilution. Newborns with hypovolemic shock from acute blood loss (eg, acute feto-maternal hemorrhage, bleeding vasa previa, extensive vaginal bleeding, fetal trauma, cord disruption, umbilical cord prolapse, and severe cord compression) may require emergency volume expansion. These newborn have features of shock like pale in color, delayed capillary refill, and/ or weak pulses. Packed red blood cells should be considered for volume replacement when severe fetal anemia is suspected. If cross-matched blood is not immediately available, then non–cross-matched, type-O, Rh-negative packed red blood cells are used. The initial dose of the selected volume expander is 10 mL/kg that needs to be given as a steady infusion over 5 to 10 minutes, followed by repeat dose if the newborn does not improve after the first dose [13, 14].

The causes of shock in preterm and term newborn includes prematurity (secondary to poor vasomotor tone, immature myocardium that is more sensitive to changes in afterload and dysregulated nitric oxide production), asphyxia, early onset sepsis (EOS), air leak syndromes, myocardial dysfunction, hypovolemia, maternal anaesthesia, fetal arrhythmias and fetal blood loss (ante-partum hemorrhage, feto-maternal hemorrhage or twin to twin transfusion syndrome) [123]. Arterial blood pressure (BP) is the most frequently monitored indicator of neonatal circulatory status but studies has shown that systemic perfusion shows poor correlation with BP [124, 125]. Groves et al. showed that in infants with reduced systemic perfusion, BP tends to have normal or high values in the first hours of life and low BP didn’t correlated with poor perfusion in the first 48 h of postnatal life in sick preterm infants [126]. Lactate measurement can be done in blood gas and it is used as biomarker in diagnosing and assessing the severity of systemic hypoperfusion and can help in earlier diagnosis of shock in normotensive neonates during golden hour [127].The neonatologist should identify shock in compensated phase and should manage it aggressively. The management includes establishing early intravenous access, judicious use of fluid resuscitation, vasopressors (dopamine or dobutamine) and other supportive care like blood transfusion for hemorrhage, and antibiotics for septic shock [122, 128].

ELBW/EGLAN and VLBW are prone to have IVH during postnatal life, and of the total IVH 50% take place on day 1 of post-natal life. Indomethacin prophylaxis has been used to reduce incidence of IVH in premature neonates [129]. Trial of Indomethacin prophylaxis in preterms (TIPP) trial which is the largest study conducted to see the effect of Indomethacin prophylaxis in ELBW reported that prophylactic indomethacin lead to the reduction in the incidence of PDA, PDA ligation, IVH (grades 3 and 4), and pulmonary hemorrhage [16, 18], but there was no reduction in the incidence of death or neurodevelopmental abnormalities [130]. Cochrane meta-analysis that included 19 trials concluded that prophylactic indomethacin reduced the incidence of symptomatic PDA, PDA surgical ligation and incidence of severe IVH but there was no effect on mortality or on a composite of death or severe neurodevelopmental disability assessed at 18 to 36 months old [131]. Hence, indomethacin is not recommended for routine prophylaxis against IVH. However, indomethacin is still being used in some neonatal units depending on clinical circumstances and personal preferences [132].

As traditional clinical and biochemical markers of perfusion have little importance in the neonatal population, therefore bed side functional echocardiography (FE) has come in picture and is used as point of care in the golden hour to find out the cause of shock and help in the management of neonatal shock. FE helps in the assessment of function of the circulatory system rather than detailed anatomy and help in rapid decision making based on real time images of central blood flow [133, 134]. Assessment of superior vena cava (SVC) flow using FE has shown to the best available method of monitoring central perfusion in the neonatal population and flow within the SVC has been considered as a good surrogate marker of cerebral perfusion [135, 136]. Thus SVC flow can be assessed in neonates who are in shock or having perinatal asphyxia [137, 138].

Persistent Pulmonary Hypertension (PPHN) is also seen sometimes in sick neonates during the golden hour. PPHN is due to increased pulmonary vascular resistance or supra-systemic pulmonary pressure leading to right-to-left shunting of blood across patent foramen ovale (PFO) and patent ductus arteriosus (PDA) leading to hypoxemia [139]. PPHN is result of either maladaptation of lung parenchyma, maldevelopment of pulmonary vasculature, underdevelopment of pulmonary vasculature or intrinsic obstruction in the pulmonary vasculature. The clinical presentation of PPHN includes labile saturations, hypoxemia, high Fio2 requirement, predominant tachypnea, saturation difference (>5-10%), or PaO2 differences (10–20 mmHg) between right upper limb and lower limbs. Echocardiography is gold standard to confirm the diagnosis, and should be performed when suspicion of PPHN is there. Right to left shunting across PDA and PFO, flattening or left deviation of the interventricular septum and tricuspid regurgitation are suggestive of PPHN [140]. The management of PPHN includes optimal oxygenation, avoiding respiratory and metabolic acidosis, normoglycemia, normal metabolic milieu, blood pressure stabilization, sedation, inhaled or intravenous prostacyclin, intravenous prostaglandin E1, pulmonary vasodilator therapy (selective like Nitric Oxide and non –selective like Sildenafil and Milrinone) and extracorporeal membrane oxygenation (ECMO). Left to right shunting at both PDA and PFO level in the golden hour should be managed with optimal lung recruitment (providing adequate PEEP) and surfactant if there is parenchymal disease. Left to right shunting at PDA and right to left shunting at PFO level indicates ductal dependent right sided heart lesion and needs Prostaglandin E1 infusion, whereas vice-versa shunting across PFO and PDA shows ductal dependent left sided heart lesion and needs Prostaglandin E1 infusion and Milrinone [141–143].

### Support for nutrition

In-utero placenta provides necessary nutritional support to the fetus and as the umbilical cord is cut, the supply of nutrition is also interrupted. This makes providing support to nutrition for both term and preterm newborn a priority. In case of term newborn with no contraindications of feeding, breast feeding should be started within half hour of birth. NRP 2015 and Baby Friendly Hospital Initiative (BFHI) guidelines recommend stable newborn babies to be kept in SSC contact with mother immediately after birth and breast feeding should be done within the first half-hour following birth [13, 14, 144]. In ELBW, VLBW, EGLAN or term neonates, whom immediately feeding cannot be started, nutritional requirements needs to be taken care of. The fluid requirement of the newborn will depend upon the gestational age, and sensible/insensible water loss. The goal of fluid replacement is to provide adequate calories, protein and lipids; and compensate for the ongoing fluid losses [145]. In newborn infants the starting fluid on day 1 for birth weight < 1000 gram is 100-150 ml/kg/day, 1000-1500 gram is 80-100 ml/kg/day and for > 1500 gram is 60-80 ml/kg/day. Fluid restriction is done when there is decreased weight loss (<1%/day or a cumulative loss <5%), decreased serum sodium in the presence of weight gain (Na < 130 meq/dl), decreased urine specific gravity <1.005 or urine osmolality <100 mosm/L and increased urine output (>3 ml/kg/hr) [146]. The results from various studies have shown that restricted water intake has a beneficial effect on the incidence of PDA, BPD, NEC and death [147]. Hyponatremia with weight loss suggests sodium depletion and needs sodium replacement. Hyponatremia with weight gain suggests dilutional hyponatremia and requires fluid restriction. Hypernatremia with weight loss suggests dehydration and management includes fluid correction over 48 hours. Hypernatremia with weight gain suggests salt and water load and needs fluid and sodium restriction [148]. The daily protein and lipid requirement in ELBW/VLBW neonates is around 4-4.5 gm/kg/day and 3 gm/kg/day respectively [149–151]. The venous access should be secured in these newborn and intravenous fluids should be started at earliest to prevent hypoglycemia. The VLBW/ELBW/ELGAN newborn should be started on total parenteral nutrition (TPN) (dextrose, lipids and protein) with in the first hour of post-natal life [152–154]. The stable preterm who don’t have any contraindication of enteral feedings should be started on enteral feeds within golden hour with preference to mother milk or donor human milk [155]. The newborn who are born with surgical conditions like gastrochisis and omphalocele needs stabilization in delivery room. Sterile silastic bowel bags and/or saline-soaked gauze dressings are used to prevent damage to the exposed intestines. Handling of the bowel is minimized to prevent vascular compromise. A nasogastric tube is placed to decompress the stomach and bowel. Such newborns are sometimes born with clinical features of shock, thereafter fluid resuscitation with isotonic solutions such as normal saline or Ringer's lactate is recommended. Maintenance fluids are started (2-3 times of normal maintenance) to compensate for increased fluid loss and third space deficit. Broad-spectrum antibiotics are begun prophylactically. Any metabolic acidosis is corrected, and urgent surgical consultation is obtained [156].

The newborn who are at high risk for developing hypoglycemia after birth are premature, intrauterine growth restricted [157], sick, low birth weight, infant of diabetic mother, late preterm, large for gestational age and birth asphyxia. The neonates who are sick and have risk factor to develop hypoglycemia should get glucose level measured in golden hour [158]. The goal is to keep glucose level 50 -110 mg/dl and hypoglycemia should be managed with feeding, or dextrose infusion (glucose infusion rate (GIR) is calculated and infusion is started at GIR of 4-6 mg/kg/min in preterm and 6-8 mg/kg/min in term neonates and increased gradually to 12 mg/kg/min) as per the clinic condition of the newborn and symptoms of hypoglycemia [159, 160]. Symptomatic hypoglycemia (irritability, stupor, jitteriness, tremors, apathy, episodes of cyanosis, convulsions, intermittent apneic spells or tachypnea, weak and high pitched cry, limpness and lethargy, difficulty in feeding, and eye rolling) or glucose level < 25 mg/dl needs to be managed with bolus of 10% dextrose at 2ml/kg followed by continuous glucose infusion at GIR of 6-8 mg/kg/min. Blood sugar should be checked again after 20-30 minutes after bolus and then and then hourly until stable, to determine if additional bolus is required [161–163]. The infants who generally need GIR > 8mg/kg/min are usually severe IUGR or else having congenital hypopituitarism, adrenal insufficiency, hyperinsulinemic states, galactosemia, glycogen storage disorders, Maple syrup urine disease, Mitochondrial disorders and Fatty acid oxidation defect [164]. Recently oral 40% dextrose gel used in the management of hypoglycemia has shown to reduces the incidence of mother-infant separation for treatment and increased the likelihood of full breast feeding after discharge compared with placebo gel [165]. Hyperglycemia (defined as blood glucose level >125 mg/dl or plasma glucose level > 145 mg/dl) is also possible in golden hour. Hyperglycemia is managed by reducing the GIR and exogenous insulin is used when glucose values exceed 250 mg/dL despite decreasing GIR or when prolonged restriction of parenterally administered glucose would substantially decrease the required total caloric intake [166].

### Prevention of sepsis

Neonatal sepsis and prematurity are the two most common cause of neonatal mortality and morbidity [167–169]. The clinical manifestations of neonatal sepsis are varied and needs high degree of suspicion for early diagnosis of neonatal sepsis [170–175]. Many intervention are done to prevent neonatal sepsis, but the most important are hand washing and using asepsis precautions while handling the newborn [176–180]. The newborn should be handled with strict asepsis techniques starting from the time the neonatal birth. All invasive procedures like umbilical line or peripheral cannula insertion, administration of surfactant, preparation of IV fluids, TPN, and antibiotics should be done using aseptic precautions and bundle approach should be used [181–184]. CPAP/ventilator tubing should be sterilized and sterile distill water should be used for humidification. The newborn whom antibiotics needs to be started secondary to risk factor for early onset sepsis (EOS), first dose of antibiotic need to be given as per the unit policy in the golden hour and blood culture should be sought using aseptic precautions [1]. The risk factor for EOS includes leaking per vaginum > 18 hours; maternal features of chorioamnionitis like maternal fever, maternal tachycardia, maternal leukocytosis, foul smelling liquor, uterine tenderness, and fetal tachycardia; ≥ 3 clean vaginal examination or single unclean examination; and maternal urinary tract infection in last two weeks [185]. Placental pathological examination has also been used to confirm histologic chorioamnionitis which is defined as presence of inflammatory cells in the fetal membranes [186, 187]. Organism can be isolated by culture or PCR of the placenta, but placental cultures may be negative, even in the presence of overt histologic inflammation, thus making role of culture in diagnosis of chorioamnionitis doubtful [188]. In developing countries the most common organism responsible for early onset sepsis are Klebsiella spp., Enterobacter spp., Escherichia coli and Coagulase Negative Staphylococci [170, 189], whereas in developed countries Group B Streptococci (GBS) is most common in term, and Escherichia coli is most prevalent among premature infants [190–192]. The antibiotic therapy should be directed toward the most common causes of neonatal sepsis, including intravenous ampicillin for GBS and coverage for gram negative organisms (Aminoglycoside) and antibiotic decision should also take into consideration local antibiotic resistance patterns [193]. The total duration of antibiotics will depend upon the clinical status, results of sepsis screen and blood culture. If the blood culture shows growth of organism, antibiotics should continue for next 10-14 days; if the blood culture is sterile, sepsis screen is normal and the neonate is clinically well, antibiotics should be stopped after 48 hours and if the blood culture is negative and the neonate is well, but the sepsis screen is abnormal, then empirical antibiotics are continued for 5-7 days [194–197]. Lumbar puncture done for cerebrospinal fluid culture, biochemical and microbiological analysis is usually not recommended as routine investigation for early onset sepsis screening and it should to be done in neonates whose blood culture is positive, infants with a strong clinical suspicion of sepsis or neonate present with seizure activity, apnea, and depressed sensorium [198]. There is no role of CRP measurement in golden hour as half-life of CRP is 24 to 48 h and it takes around 10 to 12 h for level to increase, thus making CRP measurement in golden hour unreliable [199].

Fungal prophylaxis has shown to cause significant reduction in incidence of invasive fungal infection in very preterm or VLBW infants [200]. All ELBW/VLBW neonates can be started on fluconazole prophylaxis within golden hour provided the incidence of fungal sepsis is significant in the neonatal care unit (>5% at baseline) [201]. On the other hand European guidelines suggest that 2% incidence of fungal sepsis should be the threshold for implementing a fluconazole prophylaxis [202].

Mother are also sometimes infected with various viral infection at the time of neonatal birth. Such neonates needs to be managed in golden hour as per the viral infection of the mother. The neonate who are born to mother with active herpes simplex virus (HSV) genital lesions should be evaluated at 24 hours of postnatal life with HSV surface cultures (and PCRs if desired), HSV blood PCR, CSF cell count, chemistries, and HSV PCR and serum alanine transferase and should be either started on acyclovir or observed till the results of the initial tests [203, 204]. The clinical manifestation of neonatal HSV depends upon the time of acquiring infection with HSV. In-utero or congenital HSV infected neonate presents with triad of clinical manifestations at birth a). cutaneous (active lesions, scarring, aplasia cutis, hyperpigmentation or hypopigmentation) b). neurological (microcephaly, intracranial calcifications, hydranencephaly) c). ocular (chorioretinitis, microphthalmia, optic atrophy). Neonatal HSV infection acquired during the peripartum or postpartum period manifest in three forms namely Skin, eyes and mucocutaneous disease (SEM) disease [involve skin, eye, or mucocutaneous membranes]; Central nervous system (CNS) disease [involve CNS and may also have mucocutaneous involvement, but no evidence of any other organ system involvement] and disseminated disease [involve multiple organ systems including the liver, lungs, adrenals, gastrointestinal tract, CNS and the skin, eyes, or mouth] [204, 205]. The infants born to mother infected with Hepatitis-B should receive Hepatitis B immunoglobulin and single-antigen hepatitis B vaccine within 12 hours of birth. The infants who are born to HIV positive mother should be either started on exclusive breast feeding or formula feeds after discussing with parents and explaining them to avoid mixed feeding. The infants also need to be started on antiretroviral (ARV) prophylaxis with either Zidovudine or Nevirapine [206, 207].

Congenital syphilis infection occur in the newborn secondary to transmission of spirochetes across the placenta during pregnancy. The risk for congenital syphilis depends on the stage of maternal infection and the stage of infection at the time of exposure during pregnancy. The infant is examined for any physical features of congenital syphilis, dark field microscopic examination is done of any suspicious lesion or body fluids, pathological examination is done of placenta or umbilical cord and infant is evaluated with standard non-treponemal serologic tests, including venereal disease research laboratory test (VDRL) or rapid plasma (RPR) test and reactive non-treponemal tests is confirmed with a treponemal-specific test. These infants need to be started on treatment with penicillin with duration and regimen of treatment depends upon the maternal treatment received, neonatal physical examination and results of neonatal serological test [207].

There is high risk of development of antibiotic resistance with the widespread use of antibiotics thus making careful and selective use of antibiotics to the highest risk patients a universal goal. Antibiotic stewardship limits the development of antimicrobial-resistant organisms and it can be done by improving use of antibiotics. Narrow spectrum antibiotics should be used, fixed protocols should be there for starting and stopping antibiotics, start antibiotic only when clinically indicated and downgrade antibiotics after seeing blood culture sensitivity pattern are few components of antibiotic stewardship [208–210].

### Therapeutic hypothermia for asphyxia

Hypoxic–ischemic encephalopathy (HIE) is encephalopathy from peripartum asphyxia with incidence of moderate-to-severe HIE being 1–3 infants per 1000 at-term livebirths in developed countries and up to 20 infants per 1000 at-term livebirths in developing countries, with worse outcome seen in severe HIE when compared to moderate HIE [211]. This difference in asphyxia incidence is because of difference in level of antenatal care received by mother. Still in developing countries, deliveries take place in nonhospital settings in absence of health care personal, thus leading to high incidence of perinatal asphyxia in these countries [212]. Term and near-term newborn, having moderate or severe asphyxia should be started on therapeutic hypothermia if they fulfill the predefined criteria’s of eligibilty [213, 214]. The eligibilty criteria for starting therapeutic hypothermia are a) birth weight ≥ 2000 gram, post-menstrual age ≥ 36 weeks, b) evidence of fetal distress or neonatal distress as evidenced by one of the following: i. history of acute perinatal event (e.g., placental abruption, cord prolapse, severe fetal heart rate abnormality); ii. pH ≤7.0 or base deficit ≥16 mmol/L in cord gas or postnatal blood gas obtained within first hour of life; iii. 10-minute Apgar score of ≤5; iv. assisted ventilation initiated at birth and continued for at least 10 minutes, c) evidence of moderate to severe neonatal encephalopathy by examination and/or aEEG (amplitude integrated EEG) as follows: i. primary method for determining neonatal encephalopathy is physical exam (indicated by lethargy, stupor, or coma). ii. If exam shows moderate or severe encephalopathy, aEEG should be performed to provide further assessment and monitoring. iii. In circumstances in which physical exam is unreliable (e.g., muscle relaxants), an aEEG should be performed to determine if there is encephalopathy. iv. Patterns on aEEG that indicate moderate or severe encephalopathy includes the following, with minimum of 20 minutes recording time: a) severely abnormal: upper margin <10 μV b) moderately abnormal: upper margin >10 μV and lower margin <5 μV c) seizures identified by aEEG [215–217]. Neurological examination and Sarnath Staging of the newborn will help in assessment of severity of neonatal encephalopathy [218]. Electroencephalography (EEG) or aEEG have been used in classification of severity of encephalopathy, identification of seizure, see effect of anti-convulsant on seizure frequency, identification of abnormal background activity and for inclusion of starting therapeutic hypothermia [219].

Lactate is produced during anaerobic metabolism during hypoxia and poor tissue perfusion. Serum lactate has been used to as early predictor of short-term outcome after intrapartum asphyxia [220]. Shah et al. reported that initial lactate levels are significantly higher in neonates with moderate-to-severe HIE as compared to those with mild or no HIE and the lactate levels took longer to normalize in neonates with moderate to severe HIE. Thus the author concluded that highest recorded lactate level in the first hour of life and serial measurements of lactate are important predictors of moderate-to-severe HIE [221]. In other study it was shown that high lactate level after 72 hours of therapeutic hypothermia is associated with poor neurodevelopmental outcome [222]. Moderate hypothermia (33.5 °C) for 72 h after birth is the only effective neural rescue therapy for infants born at term and near-term with moderate-to-severe HIE [223]. American academy of Paediatrics (AAP) recommends therapeutic hypothermia to be started within 6 hours of birth and continued for next 72 hours followed by gradual rewarming in next 6-8 hours [224]. Cochrane meta-analysis showed significant reduction in neonatal mortality and neurological impairment with the implementation of therapeutic hypothermia [225, 226]. Therapeutic hypothermia started within one hour of post-natal life leads to reduction in incidence of clinico-electrical seizures [227]. The neonates requiring therapeutic hypothermia should be first stabilized by providing support to respiratory and cardiovascular system if required. NRP 2015 guideline recommend that in resource abundant areas newborn born at more than 36 weeks of gestation with evolving moderate-to-severe HIE should be offered therapeutic hypothermia under clearly defined protocols [213, 214]. For resource limited countries, NRP 2015 guideline states that therapeutic hypothermia may be done if the newborn fulfills predefined criteria as defined in clinical trials and in facilities with the capabilities for multidisciplinary care and longitudinal follow-up [228]. The complications of therapeutic hypothermia that are frequently seen in neonates are increase incidence of sinus bradycardia, thrombocytopenia, subcutaneous fat necrosis, hypotension, increased fibrinolytic activity, and prolongation of prothrombin time and partial thromboplastin time tests [229, 230]. Hence goal of neonatologist in case of perinatal asphyxia is to identify the neonates fulfilling the criteria for starting therapeutic hypothermia and then starting it as early as possible, preferably in golden hour [13, 14].

### Laboratory investigation

All the necessary investigations required for the management of newborn should be done in the golden hour so that there is minimal handling afterwards and decision is taken over the management plan. The investigations needs individualization as per the newborn clinical status and ante-natal risk factors. The list of various investigations includes complete blood count, blood culture, glucose, arterial blood gas (ABG) analysis/capillary blood gas, and chest X-ray (CXR). CXR helps in differentiating the various neonatal respiratory causes of distress. The CXR finding of RDS includes low lung volume, ground glass appearance, air bronchograms, reticulogranular pattern and white out lung [120]. The CXR features suggestive of TTN are prominent central perihilar vascular markings, edema of the interlobar septae, fluid in the interlobar fissures, mild cardiomegaly, minimal pleural effusion, and hyperinflation [121]. In pulmonary hypoplasia CXR will be suggestive of low lung volume on the affected side with mediastinal shift to the same side [231]. The X-ray feature suggestive of air leak syndrome will be leakage of air from the alveoli into the extra-alveolar space like pneumothorax (air collection in pleural space), pneumomediastinum (collection of air in mediastinum), pneumopericardium (collection of air around heart in pericardium), and pulmonary interstitial emphysema (PIE) (presence of air in the lung interstitium) [232–234]. CXR in meconium aspiration syndrome typically shows diffuse, asymmetric patchy infiltrates, areas of consolidation, often worse on the right, hyperinflation and sometimes presence of air leak syndrome [235]. In case of perinatal asphyxia cord blood ABG or ABG within first hour will help us decide about starting of therapeutic hypothermia. In case of immune hydrops fetalis, necessary investigations required are total serum bilirubin, direct coombs test, reticulocyte count, and hematocrit for guiding about need for partial exchange or double volume exchange transfusion (DVET) and phototherapy. Partial exchange transfusion is indicated prior to DVET when the newborn is hydropic or anemic (hematocrit <30%) [236]. Similarly CXR in case of congenital diaphragmatic hernia will tell us about the severity of lung compromise and will help us in prognostication of neonate [237].

### Monitoring/Record

Monitoring and record keeping is an important part of golden hour. All the vital parameters of the newborn like heart rate, respiratory rate, capillary refill time, invasive or non-invasive blood pressure, saturation, and blood sugar should be monitored and recorded in the newborn case record. Near infrared spectroscopy (NIRS) is newly emerging technology in which bed side assessment is done of tissue blood flow/perfusion including cerebral, renal, and gastro-intestinal tract in neonates having perinatal asphyxia, shock, cyanotic heart disease or intestinal surgeries [238–240]. NIRS has been used in management of neonates undergoing surgical correction for complex congenital heart disease. The studies shows that low cerebral regional oxygen saturation (cRSO2) correlate with poorer neurologic outcomes and increased perioperative mortality. Interventions need to be done to increase cRSO2 if there is decrease in it by ≥ 20% from a stable baseline [241]. NIRS has been used for monitoring cRSO2 in neonates during transition after birth, a period when the brain is vulnerable to injury and dysfunction [242]. It has also been used in premature newborn to see the impact of prematurity and intensive care on early brain development [243]. The other clinical usage of NIRS includes cerebral oxygenation assessment in HIE, preterm neonates with hypotension, preterm neonates with patent ductus arteriosus (PDA), preterm neonates with respiratory distress syndrome (RDS), neonates with peri/intraventricular hemorrhage (P/IVH) and preterm neonate with apneas and bradycardias [241]. Record needs to be kept of the various intervention done with their timing as this will guide us about the scope of any improvement in the aspect of timing of various interventions in the golden hour. The records keeping includes Apgar score, interventions done during resuscitation, birth weight, axillary temperature at time of admission to nursery, time of surfactant instillation, time of umbilical catheterization, time of starting ventilation and CPAP, time of starting therapeutic hypothermia, time of giving first feeds, time of starting intravenous fluids and TPN, time of giving first dose of antibiotics, complications secondary to any neonatal procedure, size and depth of endotracheal tube, umbilical catheters and depth of feeding tube fixation [1, 237].

### Communication and counselling of family

This is an important aspect of golden hour and includes talking with parents and relatives of newborn for updating about the postnatal condition of newborn. The parents of term stable newborn, shifted to mother should be counselled regarding maintenance of temperature, frequency of breast feeding with emphasis on starting of early feeding and maintenance of asepsis in the newborn care. The parents of preterm and term newborns admitted in nursery or requiring referral to higher center, should be counselled regarding the present status, interventions that have been done till that time and further plan of management. All the questions of the parents should be answered patiently and counselling should be documented and all necessary consent should be taken from the parents like admission, procedure, transportation, and starting of hypothermia consents. The obstetrician who was involved in the antenatal care of the newborn should also be informed about the condition and plan of management [237]. Post-counselling documentation should be done and is important in providing a record to which future health-care professionals can refer like starting point for later discussions or usage as a form of legal document if advance treatment preferences are being decided [244].

### Transportation in Golden Hour

The infant may sometimes need transportation to health care center just after birth either because of lack of facilities for the neonatal care to the place neonate is born or because of home delivery to provide specific neonatal care. The aim of neonatal transport is to transfer a newborn infant requiring intensive care to a center where specialized resources and experience can be provided for the appropriate assessment and continuing treatment of a sick newborn infant [245]. These VLBW, ELBW, EGLAN and sometimes term neonates require transfer to tertiary centers for management and often hypothermic on reaching referral center due to lack of adequate precautions for hypothermia prevention during transport [246, 247]. In Golden hour, the neonate should be first stabilized and during transportation care should be taken for maintenance of temperature, sugar and necessary interventions need to be done for supporting heart, lungs and brain like giving ventilatory/CPAP support if required and starting of inotropes if the newborn is in shock. The infant should be started on intravenous fluids if shifting to tertiary health care center and referred hospital should be informed regarding this transport so that the neonate receives required care on reaching the center [248].

## Current evidence for golden hour

**Fig. 2:**
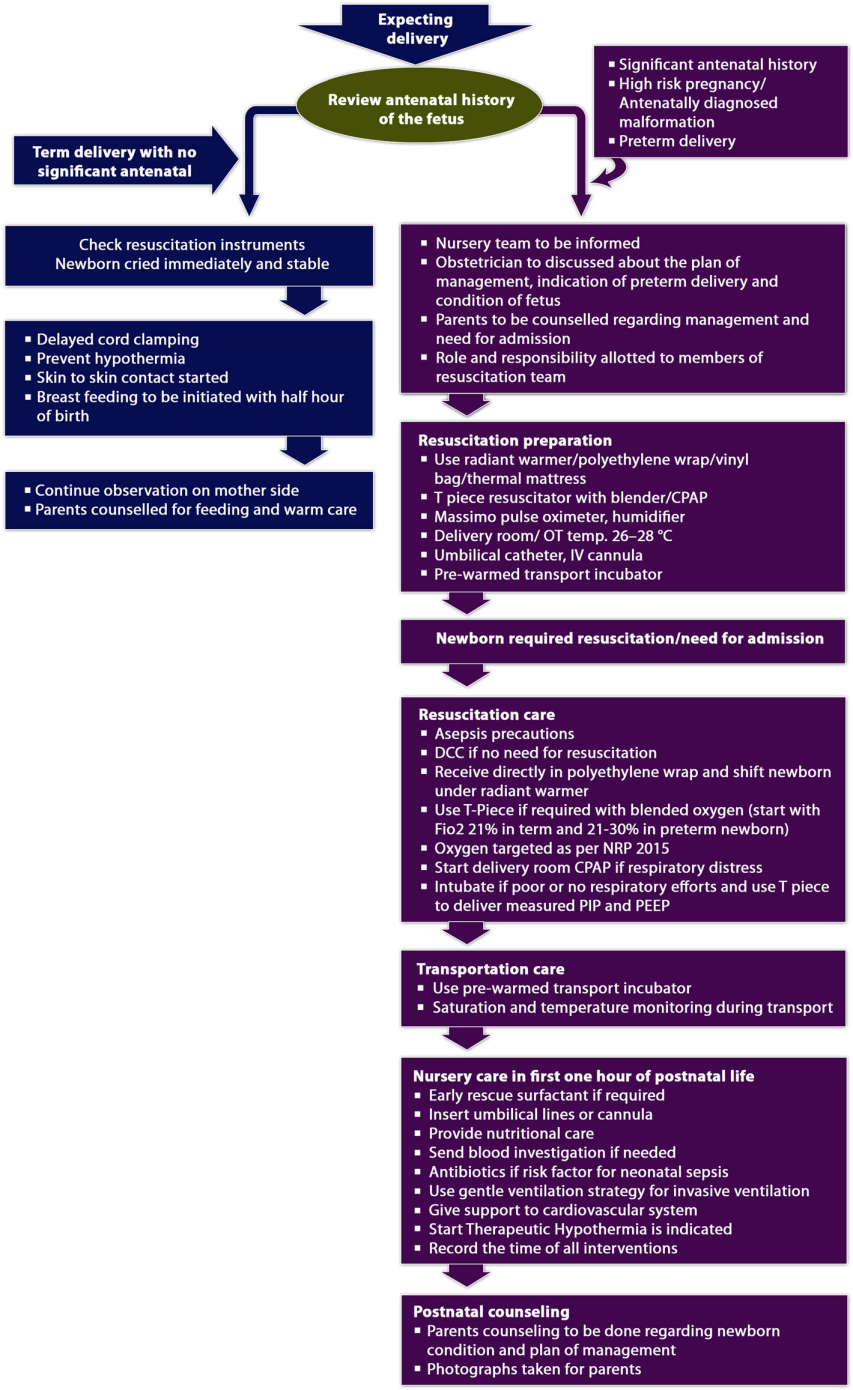
Figure showing proposed algorithm of preterm and term newborn care during golden 60 minutes of life. (Figure copyright Dr Deepak Sharma)

The current evidence that sought the effect of golden hour in care of VLBW/ ELBW reported increase in admission temperature after implementation of golden hour project [1, 249], increase in number of infants with an admission temperature of 36.5 °C – 37.4 °C [250], decrease in the incidence of ROP and BPD [1, 249], significant improvement in time of surfactant administration, time to start dextrose and amino acids infusion [7, 249, 250], significant decrease in time to give antibiotics [7], decrease in the incidence of IVH, faster placement of umbilical catheters, significant reduction in time to reach in the NICU after delivery [251], incidence of admission glucose greater than 50 mg/dL [133]. Presently, there are no studies that have sought the role of golden hour in term neonates. We purpose flow diagram for the care of preterm and term newborn during the critical golden hour that will be helpful for management of these newborns (Fig. 2).

## Conclusion

The concept of “Golden hour” is new evolving strategy for better outcome of preterm and term neonates. All the health care personal should be explained about the components and importance of golden hour approach. Neonatologist attending the birth of VLBW/ELBW/ELGAN or high-risk term neonate should be well trained in attending such deliveries and should implement all the components of golden hour during the initial first hour of post-natal life. Predelivery checklist should be followed and implemented, with teaching of the health care personal should be continuous for implementation of golden hour. The various components of golden hour include ante-natal counseling and team briefing, delayed cord clamping, prevention of hypothermia, support to respiratory and cardiovascular system, nutritional support, prevention of sepsis, therapeutic hypothermia, laboratory investigation, record keeping and counselling of parents. The current evidence supports the use of Golden hour concept by showing reduction in various neonatal morbidities like hypothermia, ROP and BPD thus showing positive impact over the survival and morbidity of the preterm and high risk term infants.

## Abbreviations

AAP: American academy of Paediatrics
ABG: Arterial blood gas
ARV: Antiretroviral
BHFI: Baby Friendly Hospital Initiative
BP: Blood Pressure
BPD: Bronchopulmonary dysplasia
CHAOS: Congenital high airway obstruction
CI: Confidence interval
CNS: Central nervous system
CPAP: Continuous positive airway pressure
CSF: Cerebrospinal fluid
CXR: Chest X-ray
DCC: Delayed cord clamping
DVET: Double volume exchange transfusion
ECC: Early cord clamping
ECMO: Extracorporeal membrane oxygenation
ELBW: Extremely low birth weight
ELGAN: Extremely low gestational age neonates
EOS: Early onset sepsis
FE: Functional echocardiography
FRC: Functional residual capacity
GBS: Group B Streptococci
GIR: Glucose infusion rate
HIE: Hypoxic–ischemic encephalopathy
HSV: Herpes simplex virus
IV: Intravenous
IVH: Intraventricular hemorrhage
NEC: Necrotizing enterocolitis
NICU: Neonatal intensive care unit
NIMV: Non-invasive mechanical ventilation
NIRS: Near infrared spectroscopy
NRP: Neonatal resuscitation programme
PDA: Patent ductus arteriosus
PEEP: Peak end expiratory pressure
PFO: Patent Foramen Ovale
PIE: Pulmonary interstitial emphysema
PIP: Peak inspiratory pressure
PPHN: Persistent Pulmonary Hypertension
PPV: Positive pressure ventilation
RDS: Respiratory distress syndrome
ROP: Retinopathy of prematurity
RPR: Rapid plasma test
RR: Relative risk
SEM: Skin, eye and mucocutaneous
SLI: Sustained lung inflation
SSC: Skin-to-skin care
SVC: Superior vena cava flow
TIPP: Trial of Indomethacin prophylaxis in preterms
TPN: Total parenteral nutrition
UCM: Umbilical cord milking
VDRL: Venereal disease research laboratory test
VLBW: Very low birth weight
